# Na_3_Zr_2_Si_2_PO_12_ Solid Electrolyte Membrane for High‐Performance Seawater Battery

**DOI:** 10.1002/advs.202300920

**Published:** 2023-04-12

**Authors:** Mengya Li, Marm Dixit, Rachid Essehli, Charl J. Jafta, Ruhul Amin, Mahalingam Balasubramanian, Ilias Belharouak

**Affiliations:** ^1^ Electrification and Energy Infrastructures Division Oak Ridge National Laboratory Oak Ridge TN 37831 USA

**Keywords:** long‐duration energy storage, Nasicon, seawater battery, sodium ion, solid‐state electrolyte

## Abstract

Seawater batteries (SWBs) have gained tremendous interest in the electrochemical energy storage research field because of their low cost, natural abundance, and potential use for long‐duration energy storage. Advancing a SWB to demonstration projects is plagued by the poor electrochemical performance stemming from the poor interfaces of the solid electrolyte (SE), as well as the structural and chemical instabilities and sluggish ionic transport properties. In this study, the anode compartment of a surrogate SWB is constructed with a Na | SE | hard carbon configuration, and tailored dopants are introduced into the Nasicon‐type Na_3_Zr_2_Si_2_PO_12_(NZSP) SE membrane. After doping with TiO_2_, a much more densely packed pellet with uniformly distributed porous structure is obtained. Changes in surface chemistry and local structure in the bulk are observed, which are believed to contribute to the improved ionic conductivity and higher critical current density of the TiO_2_‐doped NZSP. Stable cycling performance with reversible capacities based on different Na storage mechanisms are also demonstrated.

## Graphical abstract

TiO_2_ can tune the structural and chemical properties and electrochemical performances of the Na_3_Zr_2_(SiO_4_)_2_(PO_4_) solid electrolyte to meet the requirements for seawater battery applications.

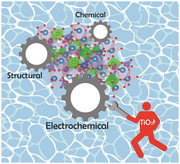

## 1 Introduction

As global warming continues to threaten the environment and ecosystems, addressing the root cause of greenhouse emissions, which mostly originate from the use of fossil fuels in transportation and industry, becomes more pressing. Global consensus suggests that the energy transition must rely on higher introduction rates of renewable sources to ensure a sustainable energy future. However, renewable energy sources such as wind or solar are intermittent in nature, and their distribution is less concentrated compared with fossil fuels. Thus, energy storage solutions are pursued to enable the wider acceptance and use of renewable energy sources in residential and commercial buildings, as well as offshore. Although most energy storage solutions suffer from deployment costs and performance issues, certain batteries suffer further from their reliance on critical materials and materials supply chain inconsistencies. Within this context, the concept of a seawater battery (SWB) recently emerged as a potential long‐duration energy storage solution because of the infinite supply of seawater.^[^ ^1^ ^]^ The implementation of seawater fits well within the global picture of net zero emissions pending the resolution of several materials and engineering challenges.

The system uses earth‐abundant seawater and its alkali metal ions, such as Na, in one chamber as the active catholyte. The Na ion is transported from the cathode chamber through a ceramic electrolyte membrane into an anode chamber containing hard carbon (HC). The overall electrochemical reaction produces a cell voltage of 3.4 V as the result of the oxidation of the alkali metal ion at the anode and oxygen reduction reaction at the cathode, forming soluble metal chlorides. The system design constantly rejuvenates reactive components, which promises long life in practical cells. An appropriate anode is essential to ensure reversible Na ion storage, and the choice of the organic electrolyte has a great effect on the electrochemical performance and practicality of the SWB. The solid electrolyte (SE), which is the separation layer between the anode and cathode compartments, plays a crucial role because it should not only promote Na transport when being in contact with nonaqueous electrolytes but should also be stable against saline water and act as physical barrier between the aqueous and nonaqueous electrolytes. Nasicon Na_3_Zr_2_Si_2_PO_12_ (NZSP) material, which has high ionic conductivity as a solid‐state electrolyte, has been proposed as a promising candidate for solid‐state Na batteries. However, solid‐state electrolytes often encounter issues such as dendrite growth and poor cycling, which ultimately lead to the failure of the cell.^[^ ^2^ ^]^ The failure mechanism is often associated with an unstable interface that can originate from the poor structural integrity, low ionic conductivity, chemical instability of the SEs, poor contact between the Na metal and SE, or a combination of more than one of these origins.^[^ ^3^ ^]^

This study focused on the SE part of the battery as doping materials such as Al_2_O_3_ and TiO_2_ were introduced in the Nasicon NZSP to tune the chemical, structural, and electrochemical properties of the SEs for the SWB. Advanced characterizations were performed to investigate the morphology, porous structure, local surface and bulk properties, and ionic conductivities of the SE NZSP. Electrochemical performance for the pristine and doped NZSP were evaluated in both a Na | NZSP | Na symmetrical cell configuration and a Na | NZSP | HC cell configuration to mimic the anode compartment of the cell. Results show that in the presence of TiO_2_ doping, NZSP could achieve higher ionic conductivity and enhanced surface stability and local structure. The TiO_2_‐doped NZSP (Ti–NZSP) also exhibited the highest critical current density (CCD) in symmetrical cells and much better cycling performance in Na | NZSP | HC cells.

## 2 Results and Discussion

**Figure 1:**
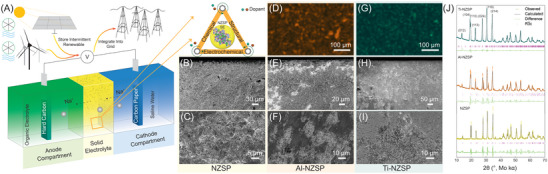
A) Schematic illustration of the seawater battery with zoom‐in scheme showing the role of dopants in this study. SEM images of B) top‐down and C) cross‐sectional views of the pristine NZSP pellet. D) Energy‐dispersive X‐ray spectrometry (EDX) elemental mapping of Al distribution within Al–NZSP from a top‐down view. SEM images of E) top‐down and F) cross‐sectional views of the pristine Al–NZSP pellet. G) EDX elemental mapping of Ti distribution within Ti–NZSP from a top‐down view. SEM images of H) top‐down and I) cross‐sectional views of pristine Ti–NZSP. J) XRD patterns and Rietveld refinement results for the pristine NZSP, Al–NZSP, and Ti–NZSP annealed at 1000 °C.

The SWB and its applications are schematically illustrated in **Figure** **1**A. The SWB is composed of an anode compartment with an HC anode in an organic electrolyte and a cathode compartment with carbon paper as the cathode in saline water (seawater) electrolyte (Figure S1, Supporting Information). The anode and cathode are separated by the SE. In the present work, Nasicon‐type NZSP was selected as the solid‐state electrolyte. To mimic the actual SWB and investigate the anode compartment, experiments were performed in Na | NZSP | Na and Na | NZSP | HC cell configurations with an organic electrolyte. By introducing different dopants into the NZSP, the chemical, structural, and electrochemical performances of the NZSP can be tuned to meet the requirements for high‐performance SEs for SWB applications. The cross section and surface of the NZSP pellet are shown in Figures 1B,C, respectively, with some pores observed at both interfaces. The scanning electron microscopy (SEM) and energy‐dispersive X‐ray spectroscopy (EDX) of the NZSP doped with Al_2_O_3_ (Al–NZSP) and TiO_2_ (Ti–NZSP) are shown in Figures 1D–F and Figure 1G–I, respectively. After doping, the density of the SE pellets increased (as can be seen from cross‐sectional SEM in Figure 1E,H). From the EDX mapping results, inhomogeneous Al distribution was observed (Figure 1D). In comparison, EDX mapping of the Ti–NZSP (Figure 1G) showed more uniformly distributed Ti dopants. A much more densely packed pellet was observed for the Ti–NZSP pellet (Figures 1H,I). The Na_3_Zr_2_(SiO_4_)_2_(PO_4_) NZSP as a Na^+^‐conducting SE material was first analyzed using a powder X‐ray diffraction (XRD) technique. The diffractogram of the material recorded in the 2*θ* range of 10°–100° is illustrated in Figure 1J. The XRD pattern confirms the high purity of the Na_3_Zr_2_(SiO_4_)_2_(PO_4_) material. All peaks are sharp and well defined, confirming the high degree of crystallinity in Na_3_Zr_2_(SiO_4_)_2_(PO_4_). Full pattern matching and refinement indicates that the material has a rhombohedral crystal structure with space group *R* $\bar{3}$ *c*, as indicated by the labeled peak positions and the magenta indexes below each pattern.^[^ ^4^ ^]^ The calculated lattice parameters for NZSP, Al–NZSP, and Ti–NZSP are summarized in Table S1 (Supporting Information). Adding 2% TiO_2_ to the pristine NZSP led to a minor modification in the lattice constants without any impurity phases. However, for NZSP with 2% Al_2_O_3_, two phases—Na_3_Zr_2_(SiO_4_)_2_(PO_4_) and NaZr_2_(PO_4_)_3_—were observed, as well as minor modification in the lattice constants. In summary, densely packed pellets and high crystallinity were achieved for NZSP, Al–NZSP, and Ti–NZSP despite the formation of some pores for the NZSP pellet and some phase separation for the Al–NZSP pellet.

**Figure 2:**
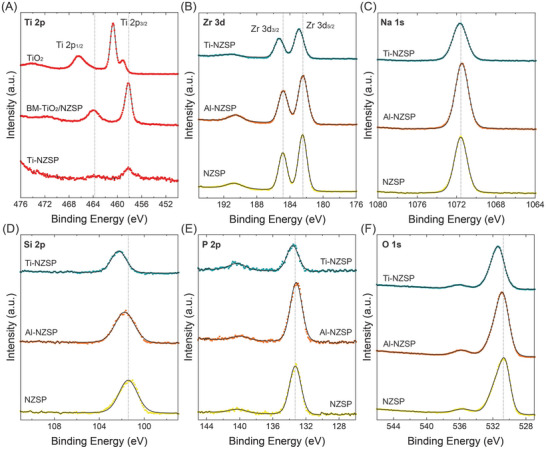
XPS results of A) Ti 2p spectra of pristine TiO_2_ powder, ball‐milled TiO_2_ powder, and pristine Ti–NZSP. For pristine NZSP, Al–NZSP and Ti–NZSP: B) Zr 3d spectra, C) Na 1s spectra, D) Si 2p spectra, E) P 2p spectra, and F) O 1s spectra (lines represent fitted results).

X‐ray photoelectron spectroscopy (XPS) analysis was performed to understand the oxidation states of each element on the surface of the samples. Titanium 2p spectra were collected for pristine TiO_2_ powder, ball‐milled TiO_2_–NZSP powder, and the Ti–NZSP pellet (**Figure** **2**A). After the ball‐milling process, the Ti 2p_1/2_ peak shifted from 467 to 464 eV, and the Ti 2p_3/2_ peak shifted from 461 to 458 eV. These peaks were maintained after the annealing process. Zr 3d spectra were collected for NZSP, Al‐NZSP, and Ti‐NZSP (Figure 2B). The Zirconium 3d_3/2_ and 3d_5/2_ peaks remained at the same binding energies (185 and 182.5 eV, respectively) after Al doping, which indicated that the Zr was in a Zr^4+^ state. However, for Ti–NZSP, the Zr 3d_3/2_ and 3d_5/2_ peaks that shifted toward higher binding energies (185.5 and 183 eV, respectively) indicated higher oxidation states were achieved for Zr. In comparison, Na 1s, Si 2p, P 2p, and O 1s spectra for the three NZSP pellets are shown in Figure 2C–F, respectively. The peak at 536 eV for O1s spectra is likely due to surface species from sample exposure to ambient environment.^[^ ^5^ ^]^ For Al–NZSP, no obvious changes in the binding energies were observed when compared with NZSP, since the introduction of Al_2_O_3_ led to a separate phase (NaZr_2_(PO_4_)_3_) that is segregated from the parent NZSP with identical (XO_4_)_3_ (X = P or Si) clusters. The binding energies for Na 1s, Si 2p, P 2p, and O 1s all shifted toward higher values for the Ti–NZSP compared with NZSP, which can be attributed to Ti doping and associated local structure changes when TiO_6_ octahedron meets the NZSP lattice. However, no strong evidence was observed of Ti^3+^ existing on the surface of Ti–NZSP based on the XPS results, which could indicate that a different compound might contain Ti^4+^ in a different local atomic arrangement, such as a distorted TiO_6_ octahedron on the surface of Ti‐NZSP.^[^ ^6^ ^]^

**Figure 3:**
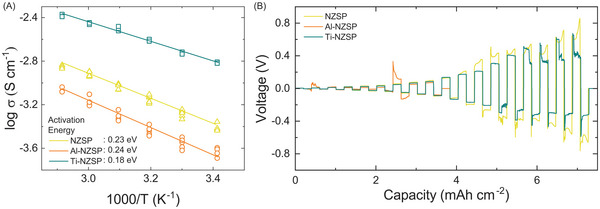
A) Ionic conductivities of NZSP, Al–NZSP, and Ti–NZSP obtained from electrochemical impedance spectroscopy measurements. B) Galvanostatic plating and stripping results at successive current densities ranging from 0.1 to 7.5 mA cm^−2^ for Na symmetric cells with NZSP, Al–NZSP, and Ti–NZSP as the SEs. Each half‐cycle corresponds to a 0.2 mAh cm^−2^ capacity.

The total conductivity of the sintered pellets was measured in blocking electrode configuration (**Figure** **3**A). The overall trend for conductivity was Ti–NZSP > NZSP > Al–NZSP, with conductivities of 1.55, 0.38, and 0.34 mS cm^−1^, respectively, at 20 °C. At 70 °C, the Ti–NZSP showed a conductivity of 4.20 mS cm^−1^ compared with 0.83 mS cm^−1^ for the Al–NZSP and 1.45 mS cm^−1^ for the NZSP. From the Arrhenius plots of the conductivity measurements, the activation energy for Na ion migration was estimated to be 0.18 eV for Ti–NZSP, 0.23 eV for NZSP, and 0.24 eV for Al‐NZSP. Transport in these crystalline materials is typically dictated by crystal structure, defect chemistry, and mobile charge concentrations. Sodium ions migrate through the tunnels made by the Si–PO_4_ tetrahedron and ZrO_6_ octahedron. Titanium doping potentially disrupts the bond lengths in this octahedron and surrounding coulombic interactions, affecting activation energy and the conductivity for ion migration. Relatively similar activation energy of the Al‐NZSP system indicates that the ion transport pathway is potentially similar to the NZSP system, although with lower conductivity owing to potential secondary insulating phases formed (as seen from the XRD patterns). Subsequently, the CCD of the SEs was measured using symmetric Na | SE | Na cells. CCD is a measure of the maximum allowable current density that can be cycled through the SE prior to filament formation.^[^ ^7^ ^]^ The protocol employed transported a 0.2 mAh cm^−2^ charge in each half‐cycle between 0.1 and 7.5 mA cm^−2^ (Figure S2, Supporting Information). The CCD was observed to follow the same behavior as the ionic conductivity: the Al–NZSP showed the lowest CCD of ≈0.05 mA cm^−2^, the NZSP failed at ≈1.4 mA cm^−2^, and the Ti–NZSP failed at ≈2.5 mA cm^−2^ (Figure 3B). The critical current values are comparable or better than the reported values for NZSP systems in the literature.^[^ ^8^ ^]^ High CCD values help in the development of systems with higher rate capabilities and are crucial for energy storage systems. Cross‐sectional image of a failed NZSP pellet in comparison with the pristine pellet is shown in Figure S3 (Supporting Information). The black region of the failed pellet indicates the formation of dendrites during CCD measurement.

**Figure 4:**
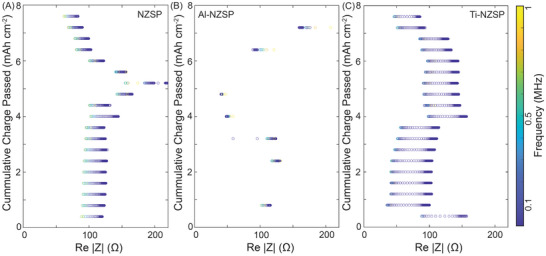
Top‐down view of electrochemical impedance spectroscopy spectra for A) NZSP, B) Al–NZSP, and C) Ti–NZSP in Na symmetric cells during CCD measurements. The length of each linear data set corresponds to the diameter of the semicircle on the electrochemical impedance spectroscopy plots.

The impedance of the symmetric cell was also tracked during cycling at the end of each plating and stripping step. A typical representation of the Nyquist diagrams is shown in Figure S4A (Supporting Information), where the *x*‐axis and *z*‐axis represent the real and imaginary part, respectively, of the impedance spectra, and the *y*‐axis is the charging condition (in this case, the amount of charge passed). For symmetric cells, the key changes in the spectra can be broadly described with two behaviors: 1) a sharp increase in the impedance consistent with physical voiding of the Na metal from the solid electrolyte interphase (SEI) and 2) a sharp decrease in the impedance consistent with the formation of filaments within the SE.^[^ ^7^, ^9^ ^]^ To visualize this change better over the duration of cell cycling, the 3D spectra was projected on the (*x*, *y*) plane so the shifts in the real part of the impedance could be easily tracked (**Figure** **4**; Figure S4B, Supporting Information). Overall, the initial impedance at the beginning of cycling was similar for all the materials. Within a few cycles, the Al–NZSP showed a very high increase in resistance (Figure S4C, Supporting Information), indicating voiding at the interface and subsequently a failure of the cell. On the other hand, the Ti–NZSP system showed a slight improvement in the impedance after the first cycle, indicating better contact at the Na | SE interface, but the NZSP impedance remained relatively consistent. After ≈4 mAh cm^−2^ of charge passed (≈1.5–2 mA cm^−2^ current density), a change in the impedance profile was seen for the NZSP and Ti–NZSP system, which might be related to Na diffusion kinetics at the applied stack pressure. Beyond that change, the NZSP system showed a sharp increase in a few cycles followed by signatures of soft shorting. Similar behavior was seen for Ti–NZSP, but it was in later cycles. The impedance spectra are consistent with the voltage polarization profiles observed within the CCD measurements, where a flat polarization is seen for the lower current densities followed by gradual sloping and nonlinear behaviors at higher current densities. Notably, this analysis is purely qualitative in nature and was carried out to assess the relative behavior of the three material systems.

**Figure 5:**
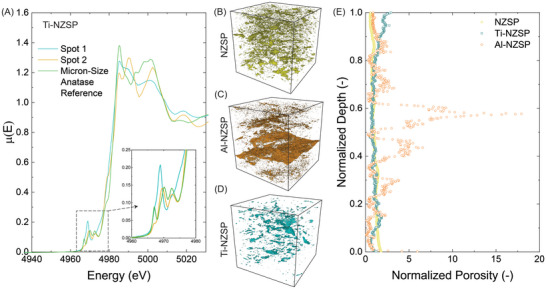
A) Ti K‐edge XANES spectra of the Ti–NZSP pellet with the micrometer‐size anatase TiO_2_ as the reference. 3D synchrotron X‐ray tomographic reconstruction of pores within the B) NZSP, C) Al–NZSP, and D) Ti–NZSP pellets (the reconstruction subvolumes are between 50 and 500 µm^3^). E) Normalized porosity versus normalized depth of the subvolumes for (B) to (D) for NZSP, Al–NZSP, and Ti–NZSP pellets.

To further characterize the bulk properties of the Ti–NZSP pellets, synchrotron X‐ray absorption near edge structure (XANES) analysis was performed on the pellet. The penetration depth was ≈12 µm at 4966 eV (Ti K‐edge). From all the spectra that were collected, at least two different Ti local structures (**Figure** **5**A) were observed. More specifically, when focusing on the pre‐edge features in the XANES spectra (inset in Figure 5A), spot 1 with a sharp high intensity peak at 4968 eV could be corresponded with a highly distorted TiO_6_ octahedron.^[^ ^10^ ^]^ This phenomenon could relate to the hypothesis proposed in Figure 2C. For spot 2, the pre‐edge XANES spectra contained three peaks near 4966, 4969, and 4973 eV, which are corresponded to quadrupolar 1s to 3d transition and dipolar 1s to Ti 3d/O 2p transitions. As a reference, the middle peak of the micrometer‐size anatase TiO_2_ standard is at 4972 eV. Thus, peak shift toward lower energy was observed for spot 2 in comparison with the standard sample, which is likely because the coordination of Ti changed from sixfold to lower.^[^ ^10^, ^11^ ^]^ Also, the XANES spectra for spot 2 looked similar to a rutile TiO_2_ standard.^[^ ^12^ ^]^ Although it is still unclear now what the exact local environment was within the Ti–NZSP structure, the authors know that changes are associated with the TiO_6_ octahedron and Ti coordination, which are a result of the introduction of TiO_2_ into the Na_3_Zr_2_(SiO_4_)_2_(PO_4_) structure.

Apart from spectroscopy results, microstructure is another key property that can affect the transport and electrochemical performance of SEs.^[^ ^7^, ^13^ ^]^ To assess the microstructure of these SEs, synchrotron X‐ray tomography was performed on the sintered pellets. Binarization of the reconstructed images enabled researchers to visualize the porosity distribution in these SEs (Figure 5B–E). Overall, the porosity of the Al‐ and Ti‐doped NZSP were slightly lower compared with the pristine NZSP material (≈1%–2% compared with 6%). However, the Al–NZSP material showed large aggregates of porosity compared with the other materials. To understand the local distribution of porosity, the X‐ray transparent region was mapped as a function of sample depth (as described previously) and normalized to the median porosity value for the material.^[^ ^14^ ^]^ The results show that NZSP and Ti–NZSP are relatively uniformly sintered, as evidenced by the near‐vertical plot of the normalized porosity. On the other hand, Al–NZSP showed large deviations from the norm with regions having porosities up to 20 times higher than the median value. It was previously shown that local heterogeneity in a microstructure can accelerate failure in SEs.^[^ ^9b^ ^]^ This study proposes that a similar mechanism in conjunction with the interphase formation leads to the reduced performance of the Al–NZSP system.

**Figure 6:**
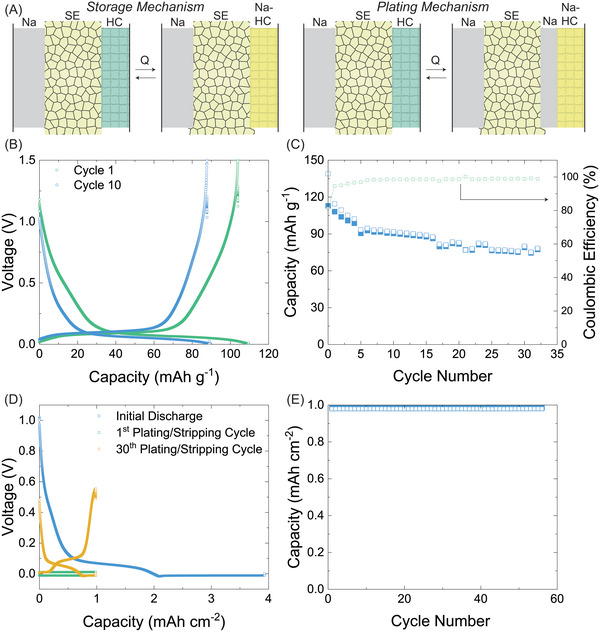
A) Schematic illustration of the storage mechanism and the plating mechanism within the Na | SE | HC cells. B) Galvanostatic charge/discharge curves and C) cycling of the Na | SE | HC cell at 70 °C between 0.005 and 1.5 V at 0.1 C (1 C = 250 mAh g^−1^). D) Galvanostatic charge/discharge curves and E) cycling performance of the Na | SE | HC cell with plating/stripping current of 1 mAh cm^−2^ and −1 to 1 V cutoff voltages.

To mimic the actual SWB system, Ti–NZSP was incorporated as solid‐state electrolyte for Na | Ti–NZSP | HC cells. By varying the cutoff voltages, this study aimed to investigate the electrochemical performances of the cell based on two different mechanisms (see schemes in **Figure** **6**A): 1) storage mechanism, where only Na ions are stored within HC and 2) plating mechanism, where Na ions are stored, and Na metal is deposited on top of the sodiated HC. The storage mechanism ensures good safety within the actual SWB but has limited energy density. The plating mechanism can ensure a system with higher energy density, and a well‐designed SE as a physical separation of the anode and cathode compartments in actual SWBs will alleviate the safety risks. For cell configurations with storage mechanisms, at C/5, an initial capacity of 110 mAh g^−1^ was delivered with a Coulombic efficiency of 81% (Figure 6B). After 30 cycles, 80 mAh g^−1^ capacity was maintained (Figure 6C). A plating mechanism, where more Na can be stored in metallic form, is beneficial for high energy applications.^[^ ^15^ ^]^ A Na | Ti‐NZSP | HC cell with the plating mechanism showed charge/discharge with a stable Na ion storage followed by reversible plating/stripping plateaus (Figure 6D). The cycling performance with 1 mAh cm^−2^ capacity involved in each cycle was more stable than it was for the storage mechanism for over 50 cycles (Figure 6E). Overall, stable electrochemical performances were demonstrated within a Na | SE |HC cell using the Ti–NZSP pellet based on both the storage and the plating mechanisms, which target different goals in actual SWBs.

**Figure 7:**
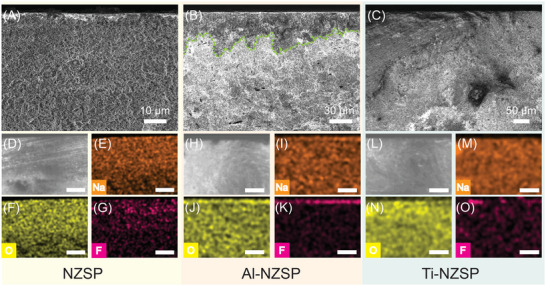
Postmortem SEM images and associated energy‐dispersive spectrometry mapping results of the cross section of failed A,D–G) NZSP; B,H–K) Al–NZSP; and C,L–O) Ti–NZSP pellets taken out of Na symmetric cells. The scale bars in (D)–(O) are 100 µm.

**Figure 8:**
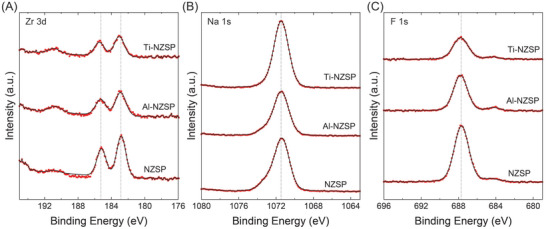
XPS results of A) Zr 3d, B) Na 1s, and C) F 1s spectra of cycled NZSP, Al–NZSP, and Ti–NZSP.

Postmortem characterizations were performed on cycled SE pellets. The SEM and EDX results of cycled NZSP, Al–NZSP, and Ti–NZSP are presented in **Figure** **7**A–C and D–O, respectively. For all the pellets, no obvious structural changes were observed for NZSP and Ti–NZSP from cross‐sectional SEM images. However, Al–NZSP developed a 30 µm thick interlayer (Figure 7B, green dotted line indicates the contrast between the interlayer and the rest of the pellet) that was rich with Na, O, and F, but not Al (Figure 7H–K; Figure S5, Supporting Information). This interlayer can be attributed to the combination of 1) the phase segregation of Na_3_Zr_2_(SiO_4_)_2_(PO_4_) and NaZr_2_(PO_4_)_3_ on the surface, and 2) the SEI formation during electrochemical testing when two drops of 1 m NaPF_6_ in diglyme were added to wet the Na | SE interface. Because of the phase separation in Al–NZSP, the SEI can be kinetically and thermodynamically unstable for Na plating and stripping, which thus explains the poor CCD value obtained for Al–NZSP in the galvanostatic plating/stripping test. XPS was also performed on cycled pellets, and the Zr 3d, Na 1s, and F 1s spectra are shown in **Figure** **8**A–C, respectively. No changes in the oxidation states were observed for Zr, Na, and F. Therefore, electrochemical cycling or interlayer formation does not change the surface chemistry of the pellets. Moreover, for the Ti–NZSP pellet, we sputtered the surface of cycled sample and compared the Ti 2p spectra of pristine, cycled and cycled then surface sputtered samples (Figure S6, Supporting Information). The results indicated that after sputtering on the cycled pellet, the positions of Ti 2p1/2 and the Ti 2p3/2 peaks remained unchanged in comparison with the pristine Ti‐NZSP sample. The results suggest that the shifting of Ti 2p peaks during the ball‐milling process not only exists on the surface but are also observed for the subsurface region.

## 3 Conclusion

In summary, a Nasicon‐type SE was developed for SWB applications. Without producing a new phase, the introduction of TiO_2_ resulted in a much denser SE and a modified NZSP surface with oxidized elements. With Ti‐doping, the ionic conductivity of the Ti–NZSP SE reached 4.20 mS cm^−1^. A high CCD of ≈2.5 mA cm^−2^ was realized in the Ti–NZSP in Na | SE | Na symmetric cell configuration. For practical application, the SWB was mimicked with a Na | SE | HC cell configuration, and it demonstrated excellent cell performances based on storage or plating mechanisms. Postmortem characterization results indicated that a much more stable SEI can be formed on the surface of Ti–NZSP. This work has provided engineering solutions toward SEs, which can pave the way for making better seawater batteries in the future.

## 4 Experimental Section

### Materials Preparation

Nasicon‐type NZSP was purchased from MSE Supplies LLC and used as is for making the baseline NZSP pellets. Mixtures of NZSP with Al_2_O_3_ and TiO_2_ (Sigma Aldrich) were prepared by ball‐milling the appropriate quantities of the powders. The doping amount of Al_2_O_3_ and TiO_2_ was set at 2 wt%. Pellets of NZSP, Al–NZSP, and Ti–NZSP were made in a hydraulic press at 5 ton at 150 °C. The pellets were subsequently sintered at 1000 °C for 24 h to obtain the final pellets. The pellets were polished through 220 to 2000 and to 3000 grit sandpapers to get a smooth, mirror‐like surface for further evaluation. The pellets used for electrochemical testing had thickness of ≈800 µm and a diameter of 10–12 mm. HC anode (KURANODE, Kuraray Co., Ltd) was mixed with carbon black (Super C65) and polyvinylidene difluoride (Solvay 9300) in a weight ratio of 92:2:6 in an *N*‐methyl‐2‐pyrrolidone solution before being casted onto Cu foil.

### Materials Characterization

The pristine NZSP powder and the sintered pellets were characterized by powder XRD. XRD patterns were collected using a Panalytical Empyrean Diffractometer using a 60 kV Mo source and 40 mA beam current. The pattern data was collected between 10°and 70° with a step size of 0.01° at room temperature. HighScore Plus software was used to analyze all the results, and the Rietveld method was adopted to refine the XRD data using Jana2006 program package software. SEM measurements of the pristine and the electrochemically cycled pellets were carried out using a Zeiss MERLIN FE‐SEM. The SEM micrographs were collected at a nominal electron excitation of 5 keV. Energy‐dispersive spectrometry mapping of these pellets was carried out at 15 keV and a working distance of 8.5 mm.

### Electrochemical Testing

Conductivity measurements of the sintered pellets were carried out using impedance spectroscopy measurements with silver blocking electrodes. Alternating current (AC) impedance measurements were carried out on a Biologic VSP 300 potentiostat between 1 MHz and 1 Hz with an amplitude of 50 mV. The impedance spectra were fit to appropriate equivalent circuits, and conductivity was estimated using the following equation

(1) $$\sigma =R\frac{t}{A}$$

where *σ* is the conductivity, *R* is the bulk resistance, *t* is the thickness of the sample, and *A* is the sample area. Conductivity measurements were performed between 20 and 70 °C, and the activation energy for the ion transport was estimated from these measurements using the Arrhenius Equation.

Symmetrical cells of Na | SE | Na were prepared in a coin cell configuration. For this configuration, NA was rolled into thin sheets and punched out to 6 mm diameter disks. Each Na | SE interface was wetted with two drops of Na liquid electrolyte (1 m NaPF_6_ in diglyme) to mimic the SWB cell. The cells were allowed to stabilize at 70 °C prior to the initiation of the CCD measurement. The cells were cycled under a fixed protocol wherein a 0.2 mAh cm^−2^ charge was cycled in each plating/stripping cycle. CCD was defined as the current density where a sharp variation from the planar deposition/dissolution profile was observed. In addition to CCD, some Na | SE | Na cells were also cycled for long‐term plating/stripping behavior. The symmetric cell was subjected to 50 cycles of 0.2 mAh cm^−2^ electrodeposition and dissolution, 50 cycles of 0.5 mAh cm^−2^ electrodeposition and dissolution, and 50 cycles of 1 mAh cm^−2^ electrodeposition and dissolution at a current density of ≈1 mA cm^−2^.

HC anodes were punched out into 6 mm disks with ≈1.7 mg cm^−2^ loading per anode. The cells of Na | SE | HC were assembled similarly as described previously. Each electrode interface was wetted with two drops of Na liquid electrolyte and assembled into a coin cell. The cells were allowed to stabilize at 70 °C prior to formation cycling. Formation cycling included 3–5 cycles at a rate of C/10 (1 C = 250 mA g^−1^) between 0.005 and 1.5 V. Subsequently, some cells were cycled within the storage region of the HC at the C/5 rate (between 1.5 and 5 mV cutoff). Alternatively, the plating stability of the Na | SE | HC cell was evaluated. After the formation cycle, the cell was allowed to discharge above the storage threshold of HC material (20 h at C/5 with −1 V cutoff). After that discharge, the cell was cycled at C/5 for 5 h between −1.5 and 1.5 V. After cycling, the cells were disassembled inside a humidity‐controlled dry room, and the SE pellets were taken out for ex situ characterization.

### X‐Ray Photoelectron Spectroscopy Studies

XPS spectra were collected on the pristine and electrochemically cycled pellets. XPS was also carried out on the pristine Al_2_O_3_ and TiO_2_ powders. All ex‐situ XPS were carried out on a SCALAB Xi+ spectrometer (Thermo Fisher Scientific) using a monochromatic Al K*α* source operated at 200 W with a 650 µm beam size. The pass energy of the spectra was 40 eV.

### X‐Ray Tomography Studies

Synchrotron tomography studies of the sintered pellets were carried out at beamline 2‐BM‐B of the Advanced Photon Source at Argonne National Laboratory. For these measurements, 3 mm pellets were separately pressed and sintered with the powders to ensure that the entire pellet could be captured in the field of view. A pink beam configuration was used to enable adequate transmission through the dense materials. In total, 1500 projections were captured during a 180° rotation of the sample with a 100 ms exposure time for each projection. A forward‐looking infrared Oryx ORX‐10G51S5M camera was used with a 2× magnification objective lens. The resultant voxel size was ≈1.7 mm with a field of view of 3.4 × 3.6 mm^2^. Under these experimental conditions, a single tomography scan took ≈7–10 min of acquisition time.

### X‐Ray Absorption Near Edge Structure (XANES) Studies

Titanium K‐edge XANES spectra were collected at beamline 20‐BM at the Advanced Photon Source at Argonne National Laboratory using a Si (111) double‐crystal monochromator, which was detuned to 15% of its original maximum intensity to eliminate high‐order harmonics. The spectroscopic data were collected using a focused beam in fluorescence mode owing to the low concentration of the element of interest (Ti) within the sample pellet. The Rh‐coated harmonic rejection mirror was set to 5 mrad to cut off the high energy harmonics. Energy calibration was performed by using the first derivative point of the XANES spectrum of Ti (K‐edge = 4966 eV) of a metallic Ti foil recorded before and after the measurement.

## Conflict of Interest

The authors declare no conflict of interest.

## Supporting information

Supporting Information

## Data Availability

The data that support the findings of this study are available from the corresponding author upon reasonable request.
