# Supplementary material for: Na3Zr2Si2PO12 Solid Electrolyte Membrane for High‐Performance Seawater Battery
Source: Adv Sci (Weinh). 2023 Apr 12;10(17):2300920. doi: 10.1002/advs.202300920 (PMC10265043; doi:10.1002/advs.202300920)
Supplement: Supplementary file 1 — Supporting Information [file ADVS-10-2300920-s001.pdf]

ADVANCED SCIENCE
Open Access

# Supporting Information

for *Adv. Sci.*, DOI 10.1002/advs.202300920

## $Na_3Zr_2Si_2PO_{12}$ Solid Electrolyte Membrane for High-Performance Seawater Battery

*Mengya Li, Marm Dixit, Rachid Essehli*, Charl J. Jafta, Ruhul Amin, Mahalingam Balasubramanian and Ilias Belharouak**

# Supporting Information

**Na3Zr2Si2PO12 Solid Electrolyte Membranes for High-Performance Seawater Battery**

*Mengya Li, Marm Dixit, Rachid Essehli,* Charl J. Jafta, Ruhul Amin, Mahalingam Balasubramanian, Ilias Belharouak**

Electrification and Energy Infrastructures Division, Oak Ridge National Laboratory, Oak Ridge, TN 37831, United States

E-mails: Rachid Essehli (essehlir@ornl.gov)

Ilias Belharouak (belharouaki@ornl.gov)

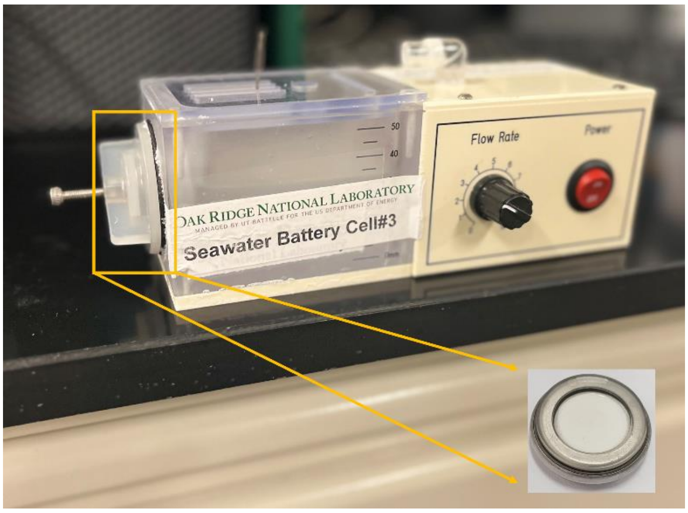


**Figure S1.** Image of the seawater battery with anode compartment highlighted.

**Table S1.** Lattice parameters of NZSP, Al–NZSP, and Ti–NZSP obtained from Rietveld refinement.

| | *a* (Å) | *b* (Å) | *c* (Å) | Unit cell volume ($Å^3$) |
|---|---|---|---|---|
| NZSP | 9.019(1) | 9.019(1) | 22.96322(1) | 1617.59 |
| Al–NZSP | 9.051(1) | 9.051(1) | 22.728(1) | 1612.40 |
| Ti–NZSP | 9.054(1) | 9.054(1) | 22.728(1) | 1613.47 |

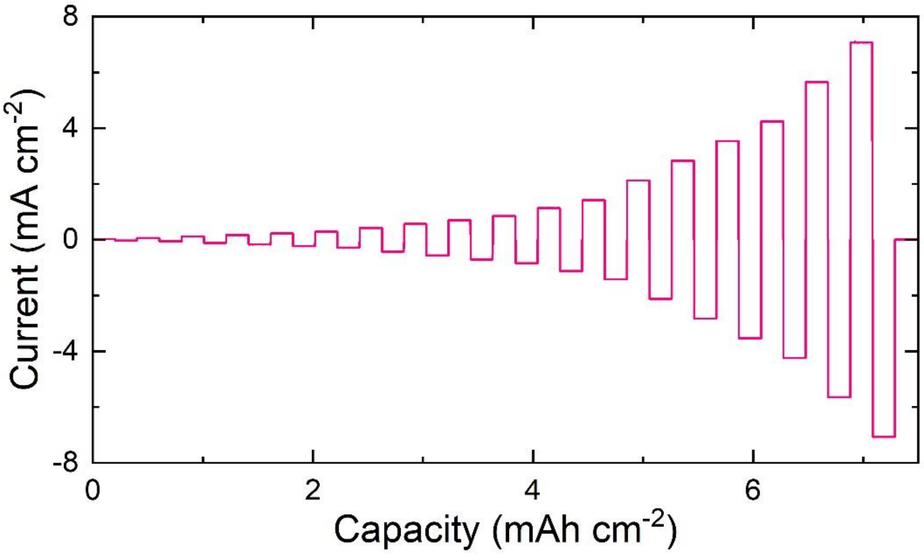


**Figure S2.** Galvanostatic plating/stripping protocol for CCD measurement for Na symmetric cells with NZSP, Al–NZSP, and Ti–NZSP as the SEs.

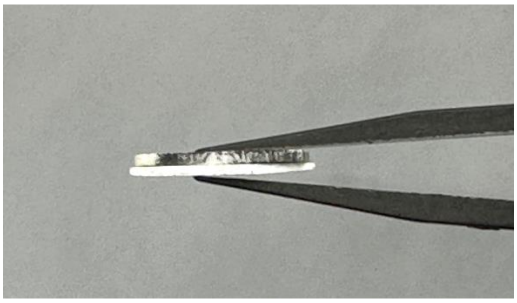

**Figure S3.** Cross-sectional image of a failed NZSP pellet (top) in comparison with the pristine pellet (bottom).

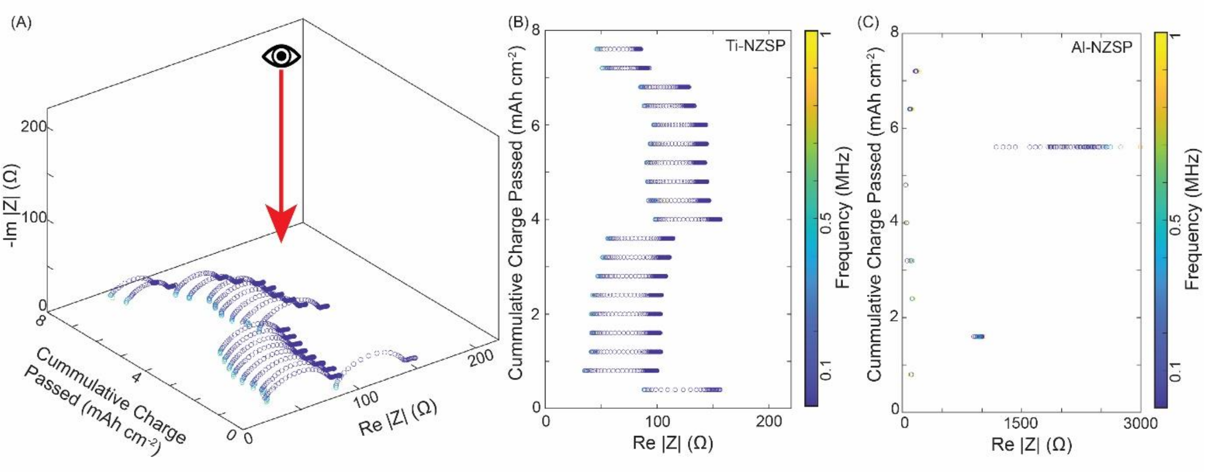


**Figure S4.** (A) Nyquist plots of Na | NZSP | Na cell during CCD measurement. Top-down view of Nyquist plots for (B) Na | Ti–NZSP | Na and (C) Na | Al–NZSP | Na cell after a few plating/stripping cycles.

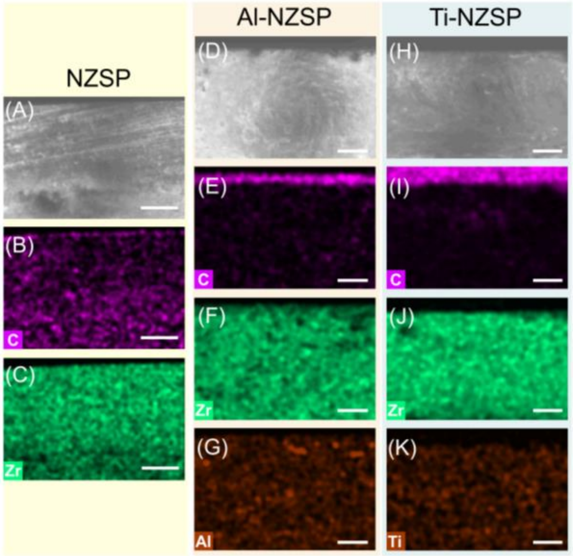


**Figure S5.** Postmortem SEM images and associated energy dispersive spectrometry mapping results of the cross section of failed (A–C) NZSP; (D–G) Al–NZSP, and (H–K) Ti–NZSP pellets taken out of Na symmetric cells. The scale bars in (A)–(K) are 100 µm.

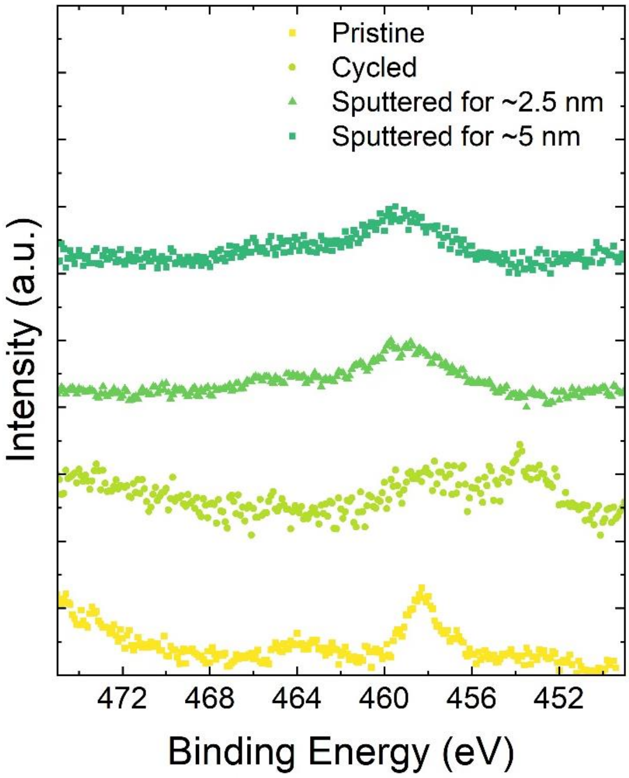


**Figure S6.** XPS results of Ti 2p spectra of pristine, cycled, cycled then sputtered for ~2.5 nm and cycled then sputtered for ~5 nm Ti-NZSP pellets.
